# In Situ Hydroxyapatite Synthesis Enhances Biocompatibility of PVA/HA Hydrogels

**DOI:** 10.3390/ijms22179335

**Published:** 2021-08-28

**Authors:** Petra Chocholata, Vlastimil Kulda, Jana Dvorakova, Monika Supova, Margit Zaloudkova, Vaclav Babuska

**Affiliations:** 1 Department of Medical Chemistry and Biochemistry, Faculty of Medicine in Pilsen, Charles University, Karlovarska 48, 301 66 Plzen, Czech Republic; petra.chocholata@lfp.cuni.cz (P.C.); vlastimil.kulda@lfp.cuni.cz (V.K.); jana.dvorakova@lfp.cuni.cz (J.D.); 2 Department of Composites and Carbon Materials, Institute of Rock Structure and Mechanics, Academy of Sciences of the Czech Republic, V Holesovickach 41, 182 09 Prague, Czech Republic; supova@irsm.cas.cz (M.S.); zaloudkova@irsm.cas.cz (M.Z.)

**Keywords:** in situ synthesis, hydrogel, scaffold, polyvinyl alcohol, hyaluronic acid, hydroxyapatite, bone tissue engineering, biological evaluation, cell viability

## Abstract

Bone tissue engineering tries to simulate natural behavior of hard tissues. This study aimed to produce scaffolds based on polyvinyl alcohol (PVA) and hyaluronic acid (HA) with hydroxyapatite (HAp) incorporated in two different ways, by in situ synthesis and physical mixing of pre-prepared HAp. In situ synthesis resulted in calcium deficient form of HAp with lower crystallinity. The proliferation of human osteoblast-like cells MG-63 proved to be better in the scaffolds with in situ synthesized HAp compared to those with physically mixed pre-prepared HAp. For scaffolds with PVA/HA/HAp ratio 3:1:2, there was significantly higher initial adhesion (*p* = 0.0440), as well as the proliferation in the following days (*p* < 0.001). It seemed to be advantageous improve the properties of the scaffold by in situ synthesizing of HAp directly in the organic matrix.

## 1. Introduction

Tissue engineering is considered as a multidisciplinary branch that interconnects a clinical medicine, cell biology, material science, and mechanical engineering. Its main objective is to improve and restore the functions of tissues [1,2]. There are created cell-seeded three-dimensional scaffolds, which are inserted into the environment of the human body. Materials used in bone tissue engineering for the scaffolds should be as similar as possible to human tissues [3,4].

Hydrogels are three-dimensional hydrophilic biopolymeric networks that can tightly absorb and retain large quantities of water or a biological fluid without dissolving in their swollen state [3,5,6]. According to the origin of the polymers of which they are composed, hydrogels can be divided into natural, synthetic or hybrid. Hydrogels can be crosslinked chemically (covalent bonds), physically (non-covalent bonds) or by combination of both [5]. The use of a scaffold in the form of hydrogels is very convenient because hydrogels are very similar to natural tissues [5] and mimic properties of extracellular matrix (ECM) [7].

Historically, the word “hydrogel” was first used in year 1804 [8], but it was an inorganic colloidal solution, which did not correspond to the modern form of hydrogels. In 1960, Wichterle and Lim developed the hydrogel as we know it today [9], and poly(hydroxyethyl methacrylate) (pHEMA) was applied as contact lenses [5,8]. The real development of various types of hydrogels for use in medicine occurs in the 1990s [9]. Since then, there has been a significant interest in the development of hydrogels, and, currently, “smart hydrogels” are widely applicated in engineering and for medical devices [8]. Hydrogels have achieved novel development since the beginning of the 21st century, and today their properties are tailored to the appropriate application [10].

In essence, we can distinguish three generations of hydrogels. The first generation is today known as water-swollen crosslinked macromolecular networks. The second generation includes temperature-sensitive hydrogels, and the third generation deals with stereocomplexation, crosslinking of hydrogels to help metal-ligands, peptide interaction, and in situ production of materials [5].

Scaffolds made of polyvinyl alcohol (PVA) and hyaluronic acid (HA) are very often used in tissue engineering. For hard tissue engineering, the disadvantage of such hydrogels is that they are too soft [11]. In the preparation of hybrid scaffolds, a combination of a natural and a synthetic polymer enriched by inorganic component were used, and the advantages and disadvantages of different phase composition were balanced, described in previous work [3]. According to our previous results, the best combination of PVA/HA/HAp is in the ratio 3:1:2. Polyvinyl alcohol (PVA), as a synthetic component, is biocompatible, elastic and hydrophilic. Unfortunately, it does not support cell attachment and due to insufficient protein absorption, biological bond formation is hindered. To avoid these shortcomings, PVA could be modified with other components [12,13].

Hyaluronic acid (HA), as a natural material, is abundantly present in the human body. Except its biocompatibility, biodegradability, and viscoelasticity, there is an advantage of HA to increase cell adhesion. That is why it could be promising material for application in biomedicine [4].

Hydroxyapatite (HAp), Ca_10_(PO_4_)_6_(OH)_2_, as an inorganic component, is a natural component of the bone. Thanks to its biocompatibility, osteoconductivity and nontoxicity, it is very often used as a replacement of hard tissue [14,15]. On the other hand, poor mechanical properties, such as brittleness and low plasticity, are its drawbacks and that is the reason to form composites of polymers enriched with HAp [14,15,16]. The most often used preparation method of these composites is just mechanical mixture of HAp and polymer. It was stated that an ordinary physical mixture can hardly approach the real bones with its properties [17]. To prepare a composite material containing HAp as similar as possible to bone apatite in morphology, it has appeared by in situ synthesis of HAp [18]. The formation of bone-like apatite demands of functional polar side groups on the surface of the organic phase, such as –COO– (in the structure of HA) and –OH (in the structure of both HA and PVA). Thanks to the negative charge of carboxylic groups, there is a greater affinity of positively charged calcium and the formation of an amorphous calcium compound which can be finally transformed into bone mineral-like apatite [14,19]. The regular arrangement of the polar groups should ensure significantly lower agglomeration of particles, and the composite material might be highly similar to the natural bone [19,20]. The method of HAp incorporation appears to affect the biological properties of scaffolds [14]. In a view of the fact that the ordinary physical mixing of HAp with an organic phase does not allow to regulate and control morphology of the prepared scaffold [21], we tried ways of making it mimic the mineralization process in the human body. In this approach, HAp was fabricated in situ, in the presence of PVA as a template serving for nucleation and growth. In situ synthesis ensures excellent particle dispersion and uniform crystallinity.

The aim of our study was to compare scaffolds based on PVA/HA with HAp incorporated by in situ synthesis with those acquired by physical mixing of prepared HAp with organic matrix. Energy dispersive X-ray spectroscopy (EDX) analysis was used to confirm the structure, Ca/P ratio, and crystallinity of both HAp types. We evaluated in vitro physical and biological properties of prepared scaffolds (swelling, degradation, hemocompatibility, and cell viability in terms of initial adhesion and proliferation).

## 2. Results

The four types of scaffolds were evaluated. A combination of PVA, HA, and HAp was used. The organic matrix was a mixture of PVA and HA in two different ratios, 3:1 and 1:1, labeled as A and B, respectively. Samples with in situ synthesis of HAp were labeled I, and samples prepared by ultrasonication were labeled U. Final samples were labeled as IHA, IHB, UHA, and UHB.

### 2.1. Analysis of HAp

**Figure 1:**
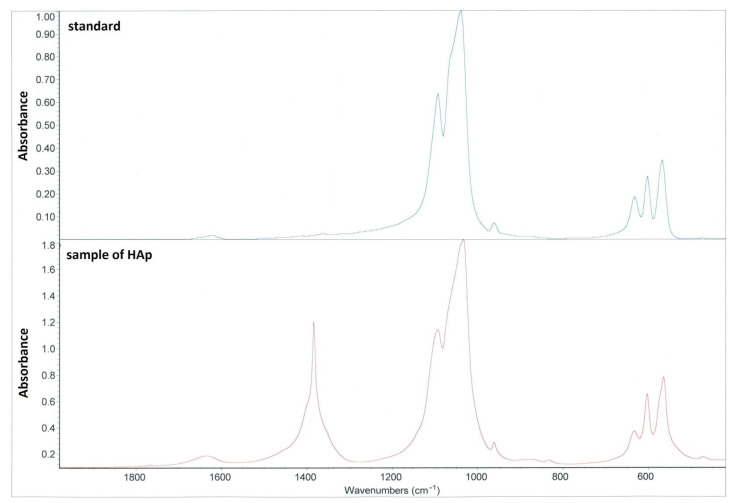
FTIR spectrum of pre-prepared HAp. Blue color—standard HAp; red color—HAp used for scaffolds. Peaks characteristic to HAp are those at 1000–1050 cm^−1^ and 560–600 cm^−1^. The additional peak at 1420 cm^−1^ marks the presence of the nitrate.

Verification of prepared HAp was performed by using Fourier-transform infrared spectroscopy (FTIR) (see Figure 1). It was obvious that the HAp produced by us corresponds to the standard. The additional peak at 1420 cm^−1^ characterizes the nitrate because HAp calcination has not been performed.

**Figure 2:**
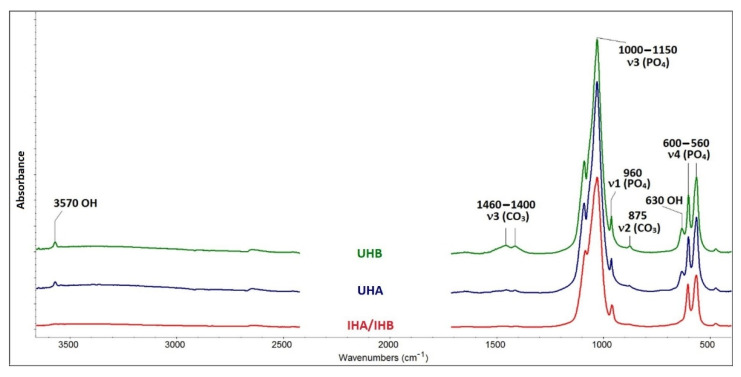
FTIR spectra. Bands in FTIR spectrum typical of valence and deformation modes of the PO_4_^3−^ anion are visible in all spectra. Bands in the range 1000–1150 cm^−1^ belong to the antisymmetric valence mode. The band at 960 cm^−1^ belongs to symmetric valence vibrations, and the bands in the range 560–600 cm^−1^ are the reflection of antisymmetric deformation vibrations. UHA, UHB—ultrasonicated; IHA, IHB—in situ prepared HAp.

For FTIR analysis of HAp in scaffolds, the materials were treated by calcination at 500 °C in air. This temperature was determined intentionally to eliminate the organic matter completely, and the inorganic part was not affected. The spectra of UHA and UHB apatites show bands at 3570 and 630 cm^−1^, which are evidence of hydroxylation of apatite in the anion channel (see Figure 2).

**Figure 3:**
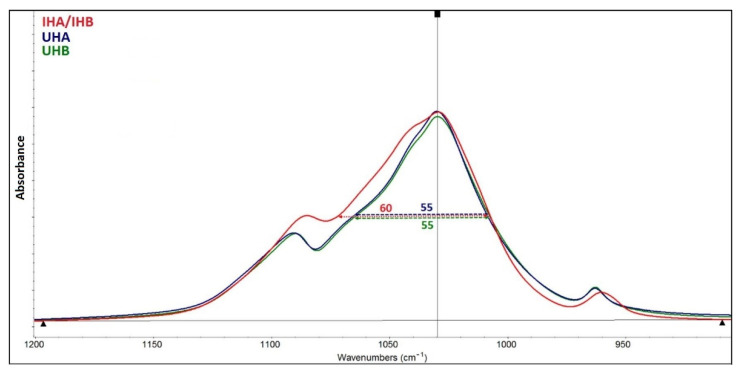
FWHM spectra. The half-width values of HAp. UHA and UHB are 55 cm^−1^, while the value for apatite IHA/IHB is 60 cm^−1^. UHA, UHB—ultrasonicated; IHA, IHB—in situ prepared HAp.

Crystallinity was measured at 1030 cm^−1^ using full width at half maximum (FWHM) approach (see Figure 3). The higher the crystallinity of the material, the lower the FWHM value is exhibited. Figure 3 shows the differences in the half-widths of all studied HAp.

**Figure 4:**
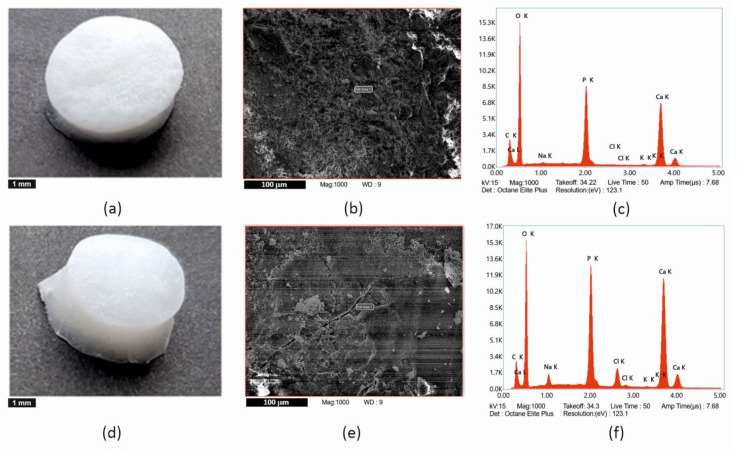
Comparison of (**a**–**c**) samples with HAp prepared by ultrasonication and (**d**–**f**) samples with HAp incorporated by in situ synthesis. (**a**,**d**) macroscopic image of the scaffolds–scale bar is 1 mm, (**b**,**e**) scanning electron microscopy images of calcinated scaffolds–scale bar is 100 µm, (**c**,**f**) spectrum of elements—EDX analysis.

The elemental composition of isolated apatites was determined by EDX (see Figure 4 and Table 1). As can be seen in Table 1, apatites contain calcium, phosphorus, and oxygen as the major elements, as well as carbon, which is part of the carbonates. Sodium and chlorine in HAp of IHA/IHB scaffolds are in concentration of approximately 2 wt%, potassium in traces. Sodium, potassium, and chlorine come from the preparation of HAp.

One of the important parameters is the weight ratio of calcium and phosphorus (see Table 2). The weight ratio of Ca/P in stoichiometric hydroxyapatite is equal to 2.15, which corresponds to a molar ratio 1.67. The performed EDX analysis showed that the Ca/P ratio in our pre-prepared HAp is approaching the value for the stoichiometric HAp (2.15). HAp prepared in situ showed a lower ratio value (1.85), which corresponds to the calcium deficient form of HAp.

To determine the effect size of the differences in the mean values of the Ca/P ratio, the Cohen’s d coefficient was calculated (see Table 3).

### 2.2. Swelling Studies

There were significant differences in swelling degree between types of scaffolds. The samples with composition ratio PVA/HA/HAp 3:1:2 (samples A) had significantly higher swelling degree than those with composition ratio PVA/HA/HAp 1:1:2 (*p* < 0.001). The results showed that the preparation method of HAp did not affect the degree of swelling. The higher the amount of hydrophilic materials, the higher the swelling degree (see Table 4).

### 2.3. Degradation Studies

Biodegradability was assessed by soaking scaffolds in simulated body fluid (SBF) for 25 days at 37 °C. All results are listed in Table 4. According to our results, the biodegradability of individual samples could not be considered significantly different, except samples with PVA/HA in ration 3:1. The ultrasonicated sample had significantly lower weight decrease than the sample with in situ prepared HAp (*p* < 0.001).

### 2.4. Hemolytic Test

All samples could be considered as hemocompatible, except scaffold with in situ prepared HAp and PVA/HA ratio 3:1, and results can be seen in Table 4. The significantly different hemolysis was recorded for samples with PVA/HA ratio 1:1 (*p* = 0.0081), and even for samples containing in situ prepared HAp (*p* = 0.0336). It should be emphasized that it was just a preliminary test; more detail and extended testing of biocompatibility must be performed.

### 2.5. Cell Viability

**Figure 5:**
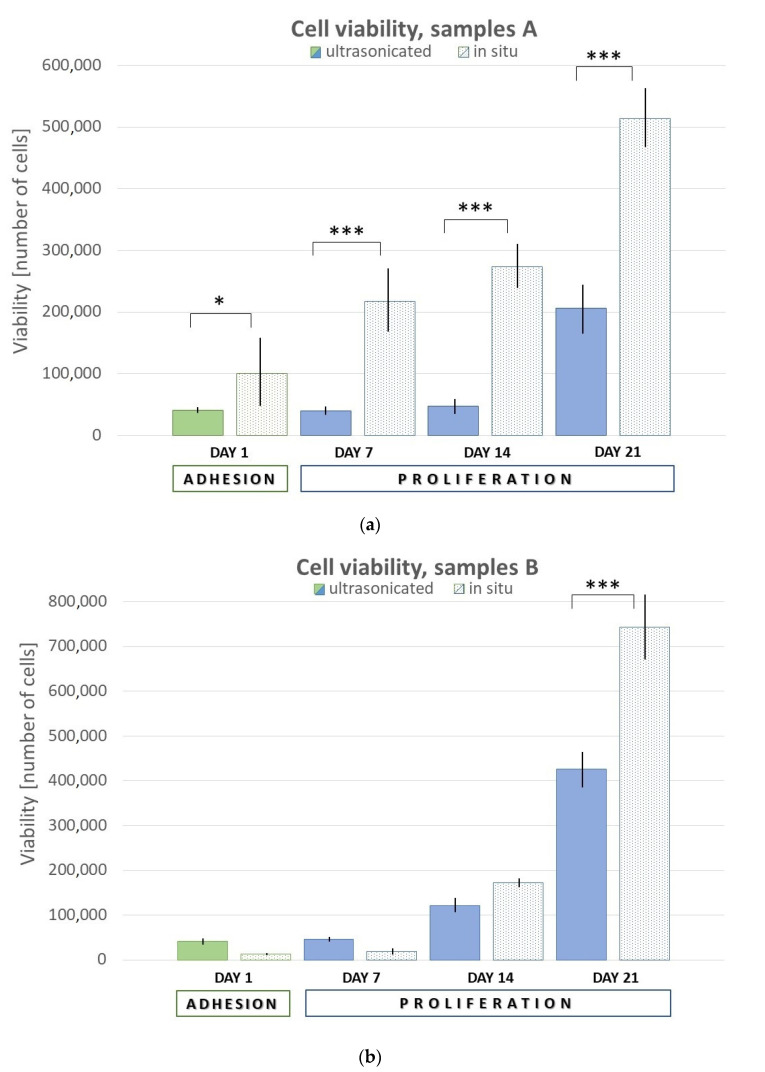
Graphical representation of adhesion and proliferation. (**a**) Samples UHA, IHA and (**b**) samples UHB, IHB. Error bars represent ±SD. Samples A—PVA/HA/HAp = 3:1:2, Samples B—PVA/HA/HAp = 1:1:2; U—ultrasonicated; I—in situ. Asterisks (*, ***) indicate statistically significant differences of *p* < 0.05 and *p* < 0.001, respectively.

Testing of cell viability on scaffolds comprised initial adhesion (day 1, 24 h post-seeding) and further proliferation (day 7, 14, and 21). The assessment was performed using Cell Counting Kit-8 (CCK-8) by measuring of increasing absorbance of formazan. Results are shown in the Figure 5. In general, the samples with in situ prepared HAp showed much better viability. For samples A (PVA/HA/HAp = 3:1:2), there was significantly higher initial adhesion (*p* = 0.0440), as well as the proliferation in the following days (*p* < 0.001). For the samples B (PVA/HA/HAp = 1:1:2), the significant difference was observed after a longer time on day 21 (*p* < 0.001).

**Figure 6:**
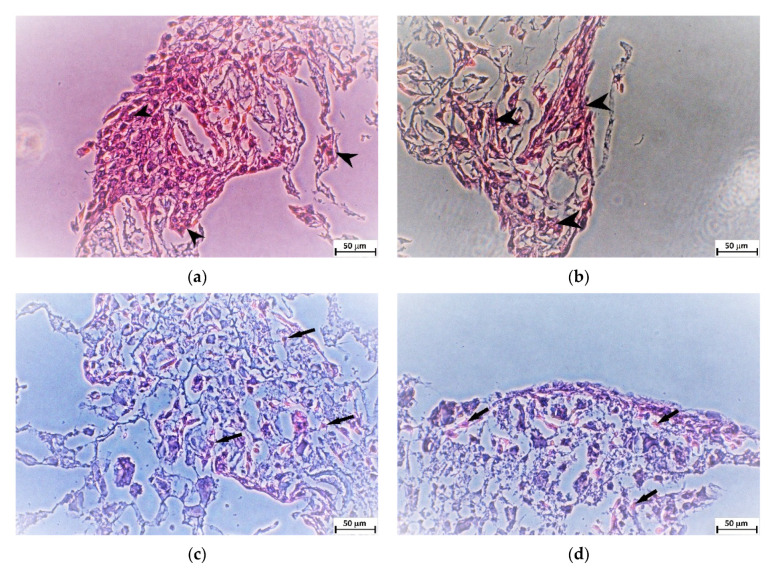
Representative microscopy images of histological slides stained with H&E showing the cells growing on the different types of scaffolds 21 days after cell seeding. (**a**) Sample IHA, (**b**) sample IHB, (**c**) sample UHA, and (**d**) sample UHB. Arrowheads indicate clusters of cells in IHA and IHB samples, while arrows point to solitaire cells in UHA and UHB samples. Scale bar is 50 µm.

Histological slides of thickness about 5 µm were prepared from scaffolds after 21 days of cell proliferation. The slides were subsequently stained with H&E. It was obvious that more cells and clusters of cells were in scaffolds with in situ synthetized HAp (IHA, IHB), while scaffolds with physically mixed ultrasonicated HAp (UHA, UHB) contained only solitaire cells (see Figure 6).

## 3. Discussion

In the preparation of hybrid scaffolds, a combination of a natural and a synthetic polymer enriched by inorganic component were used, and the advantages and disadvantages of different phase composition were balanced, described in previous work [3]. The method of HAp incorporation appears to affect the biological properties of scaffolds. Although an optimal pH is 11 for HAp formation in the bulk, in situ synthesis demand pH around 9, as Poursamar et al. found out [12]. At higher pH, there are charges more radically formed on the -OH side groups, but the absorption of Ca^2+^ ions to them is slower. On the other hand, too acidic an environment inhibits the HAp formation [16]. Ca^2+^ ions after binding on the side groups served as the centers for HAp crystals forming [22]. A local increase in Ca^2+^ ions by dissolving a less stable structure causes cell apoptosis.

Based on FTIR, a relative comparison of hydroxylation in the two HAp can be made using the ratio of the intensities of the hydroxyl and phosphate bands (I3570/I1030). The resulting values of 0.0209 for apatite UHA and 0.0275 for apatite UHB indicate that apatite UHB is more hydroxylated. The IHA/IHB material is hydroxylated only in traces. The spectrum of this apatite also lacks bands in the range of 1400–1460 cm^−1^ and a band of 875 cm^−1^, which can be assigned to the mode of valence and deformation vibration of the carbonate anion. Wavelength values below 1500 cm^−1^ indicate a substitution of type B, which means substitution of planar anions CO_3_^2−^ for tetragonal anions PO_4_^3−^, not substitution of type A, when CO_3_^2−^ anions are substituted for OH^−^ anions in the anion channel [23]. In the spectra of apatites UHA and UHB, it is also possible to make a relative comparison of carbonate substitution, using the ratio of the intensities of the bands of carbonates and phosphates (I1460/I1030). The resulting values of 0.01 for apatite UHA and 0.04 for apatite UHB indicate that apatite UHB is more substituted with carbonates. The mentioned differences in the occurrence of hydroxyl and carbonate anions in the apatite IHA/IHB and in the other two (UHA and UHB) are related to different preparation methods. UHA and UHB apatites were prepared by precipitation with solutions of Ca(NO_3_)_2_ and KH_2_PO_4_ salts at pH = 10. At such a high pH, the equilibrium in solution is adjusted in favor of the PO_4_^3−^ and hydroxyl anions [24]. The alkaline pH also allows the precipitation of CO_3_^2−^ anions, which have a strong affinity for the calcium present in the precipitate. CO_3_^2−^ anions in solution are formed from dissolved CO_2_ from air. Slight differences in hydroxylation and carbonate content in apatites UHA and UHB may be related to local inhomogeneities in solution in the experiment, e.g., the rate of stirring or dropping of one reaction solution into another can cause local changes in pH equilibrium or concentration of certain ions. Other effects may be: the experiment is performed on another day, under different atmospheric conditions, etc. Apatite IHA/IHB was precipitated on the organic matrix by a biomimetic approach using solutions of CaCl_2_ and NaH_2_PO_4_ salts at pH = 9. Lower pH records less hydroxylated forms and lower concentration of CO_3_^2−^ anions in solution and the formation of so-called calcium-deficient apatites [25]. Another factor here is the organic phase itself, which is the determining element in precipitation, e.g., what chemical groups it can provide for precipitation, and how it can affect the pH balance in solution. It also affects the size and shape of the particles, as well as the crystallinity of the precipitated apatite. Hydroxyapatite in scaffolds IHA/IHB has lower crystallinity. Apatite with lower crystallinity is better “soluble” by osteoclasts and, thus, records better bone remodeling. In the case of apatites in general: the more hydroxylated apatite, the higher its crystallinity [26], which is confirmed by this study.

With a reliability higher than 95%, we can confirm that there is probably a difference between the mean values of the Ca/P ratio of apatites UHB and IHA/IHB, similarly IHA/IHB and UHA. This difference is factually significant (large effect size according to Cohen’s d). The statistical analysis shows that the apatites UHA and UHB are, with 95% confidence, identical in their Ca/P ratios, and their values (2.18 and 2.11) are close to the value for stoichiometric HAp (2.15). IHA/IHB apatite has a ratio value of 1.85, which corresponds to the calcium-deficient form of apatite.

One of the important parameters for using scaffolds in tissue engineering is the absorption of water, which is dependent on the hydrophilic character and the composition of the scaffold [4]. The higher amount of HAp caused the decrement of hydrophilicity and the incorporation of HAp into PVA matrix made the scaffolds stiffer, and their absorbency of liquid deteriorates [27]. On the other hand, hydrophilic groups of HA allow the adhesion between material and cells, thus making the surface more bioactive [28,29].

Another important property of scaffolds is their biocompatibility. Degradable polymers are known to cause a more intense immune response of the organism than non-degradable. It is, therefore, clear that the determination of degradation is one of the key properties of scaffolds, and it influences even mechanical properties of scaffolds [27]. According to Kaur et al., higher HAp concentration should cause less weight decrease as porosity decreases. HAp forms a denser structure of the scaffold in comparison with scaffold without HAp. It was found that a higher concentration of HAp increases crystallinity and, thus, reduces the degradation rate. However, too high HAp concentration increases the degradation rate due to the formation of agglomerations and imperfect distribution of HAp particles. On the other hand, the presence of HA increases the rate of degradation. Weight loss was higher with the higher amount of HA in PVA scaffold due to breaking of crosslinking between PVA and HA segments, thus forming low molecular weight compounds. It is assumed that PVA and HA are non-toxic polymers, as well as degradation product that could be considered to be non-toxic [30].

A crucial factor to integrate the scaffold into a biological system is the evaluation of cell viability. In previous work [3], we selected two types of composites that showed the best cell viability. Based on these previous results, we continued in studying of these scaffolds. It is known that the different way of HAp incorporation to the organic matrix influences the properties of scaffolds. Used polymer can offer ionizable side groups for the HAp crystallization, and this process mimics the formation mechanism of biological composites [31]. This self-assembling process creates the scaffolds in specific shapes and significantly affects their properties [17]. The higher HAp content increases cell viability [27]. This is probably because there is a larger surface area and thus more space for cells. On the other side, Fahmy et al. stated that cell viability decreased with increasing concentration of HA during the four-day test [30]. This may be due to the degradation and the release of HA in using culture media DMEM. The DMEM probably gets into the HA structure, and it becomes more viscous, which affects cell adhesion and proliferation.

Our results showed that in situ fabrication of HAp in the organic matrix generally improved the biocompatibility of the hydrogel, in terms of initial cell adhesion and proliferation.

## 4. Materials and Methods

### 4.1. Materials

Polyvinyl alcohol (PVA, *M*_W_ 145,000, fully hydrolyzed), Ca(NO_3_)_2_·H_2_O, Ca(NO_3_)_2_·4H_2_O, NaCl, NaHCO_3_, KCl, (NH_4_)_2_HPO_4_, K_2_HPO_4_·3H_2_O, MgCl_2_·6H_2_O (all Merck, Prague, Czech Republic), hyaluronic acid (HA, *M*_W_ 1,800,000, ZVC Dr. L. Hoffmann, Cítov pod Řípem, Czech Republic), CaCl_2_, NaH_2_PO_4_·2H_2_O (Penta, Praha, Czech Republic), KH_2_PO_4_ (Lach-Ner, Neratovice, Czech Republic). MgCl_2_·6H_2_O, HCl, Na_2_SO_4_, Tris (all Lachema, Brno, Czech Republic).

### 4.2. Synthesis of Hydroxyapatite

Hydroxyapatite (HAp, Ca_10_(PO_4_)_6_(OH)_2_, Ca/P = 1.67) was synthesized by method described elsewhere [32]. Briefly, KH_2_PO_4_ of concentration 0.6M was dissolved in deionized water at room temperature with stirring. An appropriate amount of Ca(NO_3_)_2_ to keep the ratio Ca/P = 1.67 was added, to retain pH 10 using NH_3_. After stirring for an hour, the mixture was left to mature for 24 h at room temperature. To keep mixture at neutral pH, NH_3_ was removed by deionized water. The HAp slurry was dried in an oven at 70 °C for 48 h. A mortar and pestle were used to obtain final powder of HAp. The verification was performed using IR spectroscopy (FTIR spectrometer Nicolet iS5, ThermoFisher Scientific, Waltham, MA, USA).

### 4.3. Preparation of Scaffold with Pre-Prepared HAp

Preparation of PVA/HA scaffolds enriched with HAp was described in previous work [3]. Briefly, PVA solution (5%) was prepared by dissolving of PVA powder in deionized water with stirring at 90 °C until a homogenous solution was obtained. Aqueous solution of HA (1%), was prepared by dissolving of HA in deionized water with stirring at 75 °C until a homogenous solution was obtained. The mixtures of PVA and HA were prepared in volume ratios 3:1 (labeled as A) and 1:1 (labeled as B), stirred, and slightly heated (around 40 °C). Aqueous solution of HAp (5%) was added to these two types of mixtures in volume ratio HA/HAp 1:2 (labeled UHA, UHB) and ultrasonicated until homogenous blend was obtained.

### 4.4. Preparation of Scaffold with In Situ Synthesis of HAp

In situ synthesis of HAp particles was carried in presence of PVA and HA mixtures in volume ratio 3:1 and 1:1, as mentioned above. The pH around 9 had to be retained by NH_3_. At first, 0.5 M solution of CaCl_2_ and 0.3 M solution of NaH_2_PO_4_·2H_2_O in distilled water were prepared. The stock solution of calcium was slowly added to PVA/HA mixtures, and it was blended on the magnetic stirrer for 24 h at 45 °C. After that, NaH_2_PO_4_·2H_2_O stock solution was added gradually to the above mixtures, and a milky white coloration was observed, which confirmed the formation of HAp. Two types of mixtures in volume ratio HA/HAp 1:2 (labeled IHA, IHB) were prepared and were aged for 72 h at room temperature [14].

### 4.5. Preparation of Hydrogel

Both mixtures of scaffolds with HAp were poured into 24-well plates and immediately frozen at −20 °C overnight. The final porous hydrogel was obtained by thawing a frozen solution at room temperature for 12 h (1 cycle), and this procedure was repeated another 6 times, for 7 cycles in total. Cylindrical hydrogel samples with a diameter of 1.5 cm were cut to a thickness about 5 mm. Samples were sterilized by immersion into 70% ethanol for 3 h. After the sterilization, scaffolds were washed in phosphate buffered saline (PBS) and treated in culture medium at 37 °C under 5% CO_2_ in a humidified incubator overnight (approximately 12 h) to promote protein adsorption [33].

### 4.6. Analysis of Hydroxyapatite

The phase analysis of HAp was performed by Fourier-transform infrared spectroscopy (FTIR). Infrared spectra were measured by iS50 spectrometer (Thermo Nicolet Instruments Co., Madison, WI, USA) in the spectral region 4000–400 cm^−1^ with a resolution of 4 cm^−1^ by averaging of 64 scans using the ATR method. The measured spectra were evaluated using the OMNIC Software 2019 program (ThermoFisher Scientific, Waltham, MA, USA).

For energy dispersion analysis (hereinafter EDX analysis), the samples in the form of powder were adjusted to an aluminum stab using a spectral carbon target and covered with a layer of carbon on a Leica EM ACE600 sprayer (Specion s.r.o., Prague, Czech Republic). The coating thickness was 11.19 nm. EDX analyzes were measured in high vacuum mode using STEM Apreo S LoVac scanning electron microscope (ThermoFisher Scientific, Waltham, MA, USA) in an APEX EDAX system with an Octane Elite SDDs EDX detector (Ametek, Berwyn, PA, USA). Five randomly selected fields were measured at a magnification of 1000× (area 97,767 µm^2^), and the total measured area on one sample was 488,835 µm^2^. An accelerating voltage of 15 kV and the correction for carbon coverage was set to 11.2 nm.

### 4.7. In Vitro Biological Evaluation of Hydrogels

#### 4.7.1. Swelling Studies

The degree of swelling was estimated in the same way as in previous work [3]. Freeze-dried samples were soaked into 10 mL of PBS solution at 37 °C and weighted up to invariable weight. The swelling degree (SW) was evaluated for three samples of each scaffold type:

SW [%] = ((m_f_ − m_i_)/m_i_) × 100, (1)

where m_i_ is the initial weigh of the sample, and m_f_ is the final invariable weight of the sample.

#### 4.7.2. Degradation Studies

Assessment of biodegradability was estimated by soaking scaffolds in simulated body fluid (SBF) for 25 days at 37 °C. The SBF was prepared according to Kokubo et al. protocol [34]. The freeze-dried samples were weighted and immersed into the tubes containing 10 mL of SBF, sealed, and incubated for 25 days at 37 °C. After that, washed scaffolds were dried in a laboratory oven at 50 °C for 4 days and weighted. The weight decrease was determined by using the equation:

weight decrease [%] = ((m_a_ − m_b_)/m_a_) × 100, (2)

where m_a_ is the initial weight of the sample, and m_b_ is the weight after incubation and drying, final weight.

#### 4.7.3. Hemolytic Test

The hemolytic test [35] could help to verify hemocompatibility. Fresh human blood (8 mL; in a test tube with sodium citrate) was diluted with 10 mL of physiological solution (0.9% NaCl). One sample was added to each pre-heated tube for 37 °C for 30 min, and 0.2 mL of diluted blood was added and heated to 37 °C for 60 min. Three discoid scaffold replicates of each type were used. The solution of 0.2 mL diluted blood in 10 mL of physiological solution was used as a negative control, and 0.2 mL of diluted blood in 10 mL of distilled water was used as a positive control. After heating, tubes were centrifuged for 5 min at 3000 rpm. Hemocompatibility was determined photometrically (UV/VIS Spectrophotometer Optizen POP Nano Bio, Mecasys Co., Seoul, South Korea) at 545 nm wavelength as a percentage of hemolysis:

hemolysis [%] = ((A_S_ − A_NC_)/(A_PC_ − A_NC_)) × 100, (3)

where A_S_ is the absorbance of the sample, A_NC_ is the absorbance of negative control, and A_PC_ is the absorbance of positive control.

### 4.8. Cell Viability Tests

#### 4.8.1. Cell Cultures

Human osteoblast-like MG-63 cell line (ECACC 86051601, Sigma Aldrich, St. Louis, MO, USA), obtained from an osteosarcoma of a 14 year old male, was cultivated in Dulbecco’s Modified Eagle’s Medium (DMEM, Biosera Europe, Nuaille, France) supplemented with 10% (*v*/*v*) fetal bovine serum (FBS, Biosera Europe, Nuaille, France), 100 U/mL penicillin, 100 mg/mL streptomycin (PAA Laboratories GmbH, Austria), and 2.5 mM stable glutamine (Diagnovum GmbH, Ebsdorfergrund, Germany), at 37 °C under 5% CO_2_ in a humidified incubator. Culture medium was refreshed as needed [36].

#### 4.8.2. Tests of Cell Adhesion and Proliferation

The samples of scaffolds in 24-well plates (TPP Techno Plastic Products, Trasadingen, Switzerland) were seeded by 1 mL of the suspension with 4 × 10^5^ cells using a syringe with a needle of diameter 0.6 mm. After 24 h post-seeding, an initial adhesion was determined by Cell Counting Kit-8 (CCK-8, Sigma Aldrich, Darmstadt, Germany) according to the manufacturer instructions. The CCK-8 assay is based on the conversion of light purple highly water-soluble tetrazolium salt WST-8 (2-(2-methoxy-4-nitrophenyl)-3-(4-nitrophenyl)-5-(2,4-disulfophenyl)-2H-tetrazolium, monosodium salt) to orange water-soluble formazan dye, which can be spectrophotometrically quantified. The amount of the formazan dye generated by the activity of dehydrogenases in cells is directly proportional to the number of living cells. Briefly, the samples were transferred to a new 24-well plate and 550 µL of premix (CCK-8 + DMEM) was added to each sample. After incubation (60 min, 37 °C, 5% CO_2_) samples were removed, and the amount of formazan was determined photometrically at 450 nm (microplate reader SYNERGY H1, BioTek, Winooski, VT, USA). The number of viable cells was estimated based on a calibration curve. The primary adhesion was calculated according to the formula

adhesion [%] = (N/N_0_) × 100, (4)

where N is the number of viable cells after 24 h post-seeding, and N_0_ is the number of seeded cells (4 × 10^5^).

**Figure 7:**
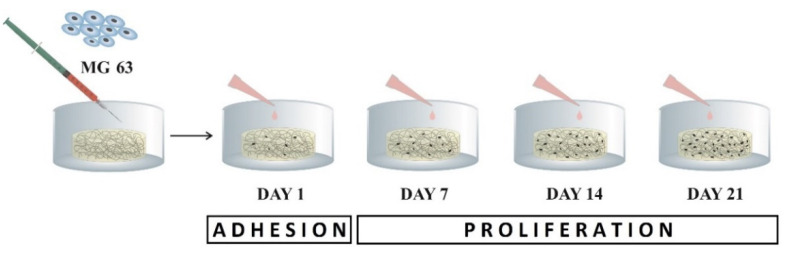
Scheme of the experiment for assessment of cell viability on scaffolds. Initial adhesion was read on day 1; further proliferation was evaluated on days 7, 14, and 21.

Cell proliferation was quantified as the number of viable cells based on a calibration curve using CCK-8, in time point day 7, 14, and 21 post-seeding (see Figure 7).

### 4.9. Histological Preparation

The samples were fixed and stored in 10% neutral-buffered formalin after day 21 of cell culturing. Standard dehydration in ethanol was performed followed by immersion in xylene, paraffin saturated xylene, and, finally, molten paraffin. Tissue blocks were cut at 5 μm (Microtom Leica RM2255, Leica Biosystems, Wetzlar, Germany) and stained by hematoxylin and eosin solutions (H&E) for cell visualization [37]. The stained slices were observed under the inverted optic microscope with a digital camera (Olympus CKX41, Olympus, Tokio, Japan).

### 4.10. Statistical Analysis

The results were estimated as the average of three samples, and each sample was measured four times. Null hypotheses about the agreement of the mean values were tested using ANOVA and post-hoc test (Student’s *t*-test). The results with *p* < 0.05 were considered statistically significant.

The statistic assessment of HAp analysis was performed in the JASP program (JASP 0.14.1, JASP Team, University of Amsterdam, Amsterdam, Netherlands). For evaluation, we assumed a normal distribution of data (Q-Q graphs), the agreement of variances was verified using Levene’s test. The null hypotheses about the agreement of the mean values were tested by ANOVA, and post-hoc tests (Student’s *t*-test) were performed with Tukey’s, Bonferroni’s, or Šidák’s correction with the original probability of occurrence of a type I error of 0.05. To determine the material effect of the differences in the mean values, the Cohen coefficient d was calculated with standard limits for its magnitude, and the standard deviation for its determination was calculated as the weighted standard deviation of both samples.

## 5. Conclusions

Preparing of scaffolds by bare physical mixing of HAp into the organic matrix prevented not only control of morphology but also the chemical interaction of HAp and organic phase. It seemed to be advantageous improve the properties of the scaffold by in situ synthesizing of HAp directly in the organic matrix. Comparing of the HAp enriched PVA/HA scaffold showed that in situ fabrication of HAp in the organic matrix improved the biocompatibility of the hydrogel.

## Figures and Tables

**Table 1:** Chemical elements composition of isolated HAp based on EDX analysis.

Element [Weight %]	IHA/IHB	UHA	UHB
Ca	33.06 ± 1.59	36.85 ± 3.11	36.40 ± 3.84
P	17.87 ± 0.15	16.87 ± 1.68	17.26 ± 1.76
O	39.80 ± 1.24	39.74 ± 4.35	39.40 ± 3.66
C	5.40 ± 0.7	6.05 ± 0.77	6.15 ± 0.58
Na	1.83 ± 0.22	0.03 ± 0.01	0.01 ± 0.01
Cl	2.03 ± 0.54	0.01 ± 0.01	0.03 ± 0.01
K	0.02 ± 0.02	0.45 ± 0.04	0.75 ± 0.08

**Table 2:** The weight ratio of calcium and phosphorus.

Weight Ratio	IHA/IHB	UHA	UHB
Ca/P	1.85 ± 0.14	2.18 ± 0.11	2.11 ± 0.12

**Table 3:** Cohen’s d coefficient of Ca/P ratio values.

Comparison	Cohen’s d Coefficient	Cohen’s d (Pair)
IHA/IHB vs. UHA	2.628	2.650
IHA/IHB vs. UHB	1.955	2.060
UHA vs. UHB	0.638	0.439

**Table 4:** Results of swelling degree, bioactivity assessment, and the test of hemocompatibility. UHA, UHB … ultrasonicated. IHA, IHB … in situ synthesis of HAp.

SAMPLE	Composition Ratio [PVA/HA/HAp]	Swelling Degree [%]	Degradation [%]	Hemolysis ^1^ [%]
UHA	3:1:2	383 ± 54	8.4 ± 0.8	18.1 ± 1.2 (++)
IHA	3:1:2	392 ± 49	16.0 ± 0.5	21.1 ± 1.0 (+)
UHB	1:1:2	211 ± 7	15.0 ± 2.7	14.7 ± 2.0 (++)
IHB	1:1:2	170 ± 24	17.2 ± 2.8	3.8 ± 0.4 (+++)

^1^ Highly hemocompatible (<5 % hemolysis, +++), hemocompatible (within 10 % hemolysis, ++), and non-hemocompatible (>20 % hemolysis, +).

## Data Availability

Not applicable.

## Abbreviations

CCK-8	cell counting kit-8
DMEM	Dulbecco’s modified Eagle’s medium
ECM	extracellular matrix
EDX	energy dispersive X-ray spectroscopy
FBS	fetal bovine serum
FTIR	infrared spectroscopy with Fourier transformation
FWHM	full width at half maximum
HA	hyaluronic acid
HAp	hydroxyapatite
pHEMA	poly(hydroxyethyl methacrylate)
PVA	polyvinyl alcohol
SBF	simulated body fluid
WST-8	water soluble tetrazolium salt
